# Salsolinol Attenuates Doxorubicin-Induced Chronic Heart Failure in Rats and Improves Mitochondrial Function in H9c2 Cardiomyocytes

**DOI:** 10.3389/fphar.2019.01135

**Published:** 2019-10-11

**Authors:** Jianxia Wen, Lu Zhang, Honghong Liu, Jiabo Wang, Jianyu Li, Yuxue Yang, Yingying Wang, Huadan Cai, Ruisheng Li, Yanling Zhao

**Affiliations:** ^1^College of Pharmacy, Chengdu University of Traditional Chinese Medicine, Chengdu, China; ^2^Department of Pharmacy, Fifth Medical Center, General Hospital of Chinese PLA, Beijing, China; ^3^College of Pharmacy, Zhejiang Chinese Medical University, Hangzhou, China; ^4^Integrative Medical Center, Fifth Medical Center, General Hospital of Chinese PLA, Beijing, China; ^5^Research Center for Clinical and Translational Medicine, Fifth Medical Center, General Hospital of Chinese PLA, Beijing, China

**Keywords:** Salsolinol, doxorubicin, chronic heart failure, energy metabolism, mitochondrial calcium uniporter

## Abstract

**Backgrounds:** Salsolinol (SAL), a plant-based isoquinoline alkaloid, was initially isolated from Aconiti Lateralis Radix Praeparata (ALRP) and identified as the active cardiotonic component of ALRP. This study was aimed to explore the therapeutic effect and mechanism by which SAL attenuates doxorubicin (DOX)-induced chronic heart failure (CHF) in rats and improves mitochondrial function in H9c2 cardiomyocytes.

**Methods:** Rats were intraperitoneally injected with DOX to establish CHF model. Therapeutic effects of SAL on hemodynamic parameters, serum indices, and the histopathology of the heart were analyzed *in vivo*. Moreover, H9c2 cardiomyocytes were pretreated with SAL for 2 h before DOX treatment in all procedures *in vitro*. Cell viability, cardiomyocyte morphology, proliferation, and mitochondrial function were detected by a high-content screening (HCS) assay. In addition, a Seahorse Extracellular Flux (XFp) analyzer was used to evaluate the cell energy respiratory and energy metabolism function. To further investigate the potential mechanism of SAL, relative mRNA and protein expression of key enzymes in the tricarboxylic acid cycle *in vivo* and mitochondrial calcium uniporter (MCU) signaling pathway-related molecules *in vitro* were detected.

**Results:** The present data demonstrated the pharmacological effect of SAL on DOX-induced CHF, which was through ameliorating heart function, downregulating serum levels of myocardial injury markers, alleviating histological injury to the heart, increasing the relative mRNA expression levels of key enzymes downstream of the tricarboxylic acid cycle *in vivo*, and thus enhancing myocardial energy metabolism. In addition, SAL had effects on increasing cell viability, ameliorating DOX-induced mitochondrial dysfunction, and increasing mitochondrial oxygen consumption rate (OCR) and extracellular acidification rate (ECAR) in H9c2 cardiomyocyte. Moreover, we found that SAL might have an effect on improving mitochondrial respiratory function and energy metabolism *via *inhibiting excessive activation of MCU pathway in H9c2 cells. However, the protective effect could be ameliorated by ruthenium red (an MCU inhibitor) and abrogated by spermine (an MCU activator) *in vitro*.

**Conclusion:** The therapeutic effects of SAL on CHF are possibly related to ameliorating cardiomyocyte function resulting in promotion of mitochondrial respiratory and energy metabolism. Furthermore, the potential mechanism might be related to downregulating MCU pathway. These findings may provide a potential therapy for CHF.

## Introduction

Chronic heart failure (CHF), the most common form of cardiovascular disease, is a serious health problem worldwide, with rising incidence and prevalence (Stephan et al., 2017; Zhang et al., 2018). According to the “Heart disease and stroke statistics—2018 update: a report from the American Heart Association” (Benjamin et al., 2018), the prevalence of CHF will increase at 46% from 2012 to 2030, resulting in more than 8 million people not less than 18 years of age with heart failure (HF). Prevention and treatment of CHF will effectively reduce morbidity and mortality rates in human beings. Currently, although various potential mechanisms have been investigated for the treatment of CHF, there is still a lack of satisfaction for the targeted therapy. The heart is the main energy-consuming organ of one’s body, and sufficient energy supply is a normal guarantee for maintaining its own needs and pumping function. Clinically, the insufficient myocardial energy supply or metabolic imbalance will lead to the abnormal structure and function of the heart and eventually lead to the occurrence of HF (Watanabe et al., 2010). In 2004 (van Bilsen et al., 2004), Van Bilsen proposed the concept of energy “metabolic remodeling,” that is, the failing heart suffers from chronic energy starvation. The changes in mitochondrial function can also cause changes in heart function. From a metabolic point of view, the hypertrophic heart is characterized by a significant change in matrix preference from fatty acids to glucose. As energy metabolism is affected during compensated hypertrophy and cardiac failure, compounds that can promote mitochondrial energy metabolism may be potential drugs for the treatment of HF (Neubauer et al., 1997; Kalsi et al., 1999).

In the prevention and treatment of HF, with the safety and multi-target advantages, traditional Chinese medicine (TCM) protects myocardial mitochondria during HF, thus providing a new idea for studying the pathogenesis and targeted treatment of CHF. Our previous study had shown that Aconiti Lateralis Radix Praeparata (ALRP, *Fuzi* in Chinese) had an effect on anti-acute HF by enhancing mitochondrial biogenesis *via* Sirt1/PGC-1a pathway (Lu et al., 2017) and anti-CHF by promoting energy metabolism *via* PPARα/PGC-1α/Sirt3 pathway (Wen et al., 2019). However, the active components of this herbal couple are still unclear. Salsolinol (6,7-dioxy-1-methyl-1,2,3,4-tetrahydroisochinoline, SAL), a plant-based isoquinoline alkaloid, was initially isolated from ALRP and identified as 1-methyl-6,7-dihydroxy-1,2,3,4-tetrahydroisoquinoline by Dihua Chen in 1982 (Chen and Liang, 1982). Due to the effect on the biosynthesis of catecholamines *in vivo*, SAL has gradually attracted the attention of researchers. Experiments have shown that SAL is a weak beta-adrenergic stimulant that excites the atrium of isolated guinea pigs and increases the frequency of contraction. It can also increase the blood pressure of normal and ruined spinal cord rats and demonstrate excitatory effects on both β- and γα-adrenergic bodies (Zhou, 2011). As a water-soluble active ingredient in aconite, SAL has a certain cardiotonic effect. However, whether SAL can promote mitochondrial energy metabolism remains to be studied.

Mitochondrial calcium ion (Ca^2+^) homeostasis plays a vital role in cellular physiological functions, including energy metabolism of cardiomyocytes and oxygen phosphorylation process (Foskett and Philipson, 2015). Mitochondrial calcium uptake 1 (MICU1) is a mitochondrial inner membrane protein that regulates the transport of Ca^2+^ through the mitochondrial calcium uniporter (MCU) and plays a role in maintaining the homeostasis of Ca^2+^ in mitochondria, thus playing a vital role in influencing cardiovascular disease (Mallilankaraman et al., 2012). The uptake of mitochondrial Ca^2+^ is primarily achieved by MCU on the inner membrane, which has highly selective and low affinity for Ca^2+^ (Penna et al., 2018). There is an interaction relationship between MICU1 and MCU. These two combine other protein molecules to form mitochondria that can also be formed as a mitochondrial unidirectional transporter complex, among which MCU is the center of this complex. There are other regulatory proteins, including MICU1, mitochondrial calcium uptake 2 (MICU2), and essential MCU regulator (EMRE) (Foskett and Philipson, 2015). Among them, MICU1 has dual regulation of mitochondrial Ca^2+^ transport (Mallilankaraman et al., 2012). MICU2 plays a key role in maintaining cardiovascular homeostasis (Bick et al., 2017). EMRE is a necessary regulatory protein for MCU. The inhibition of EMRE will reduce the activity of MCU, which can interact with MCU, as well as MICU1 and MICU2 (Alevriadou et al., 2017; Penna et al., 2018). As the core channel of the MCU complex, MCU can mediate Ca^2+^ transport and participate in the regulation of ATP production (Zaglia et al., 2017). Therefore, MCU and the regulatory protein MICU1 play an important role in cardiomyocyte function by regulating mitochondrial Ca^2+^ uptake. Our previous study had shown that ALRP had an effect on anti-acute HF by enhancing mitochondrial energy metabolism pathway mediated by MCU (Zhang et al., 2017). However, the active components of this herbal couple are still unclear. This study was designed to investigate whether the active ingredient SAL in ALRP can protect doxorubicin (DOX)-induced H9c2 cardiomyocyte injury by affecting mitochondrial respiratory function and energy metabolism and, further, to explore whether its potential mechanism of action is related to the regulation of MCU protein expression.

**Figure 1:**
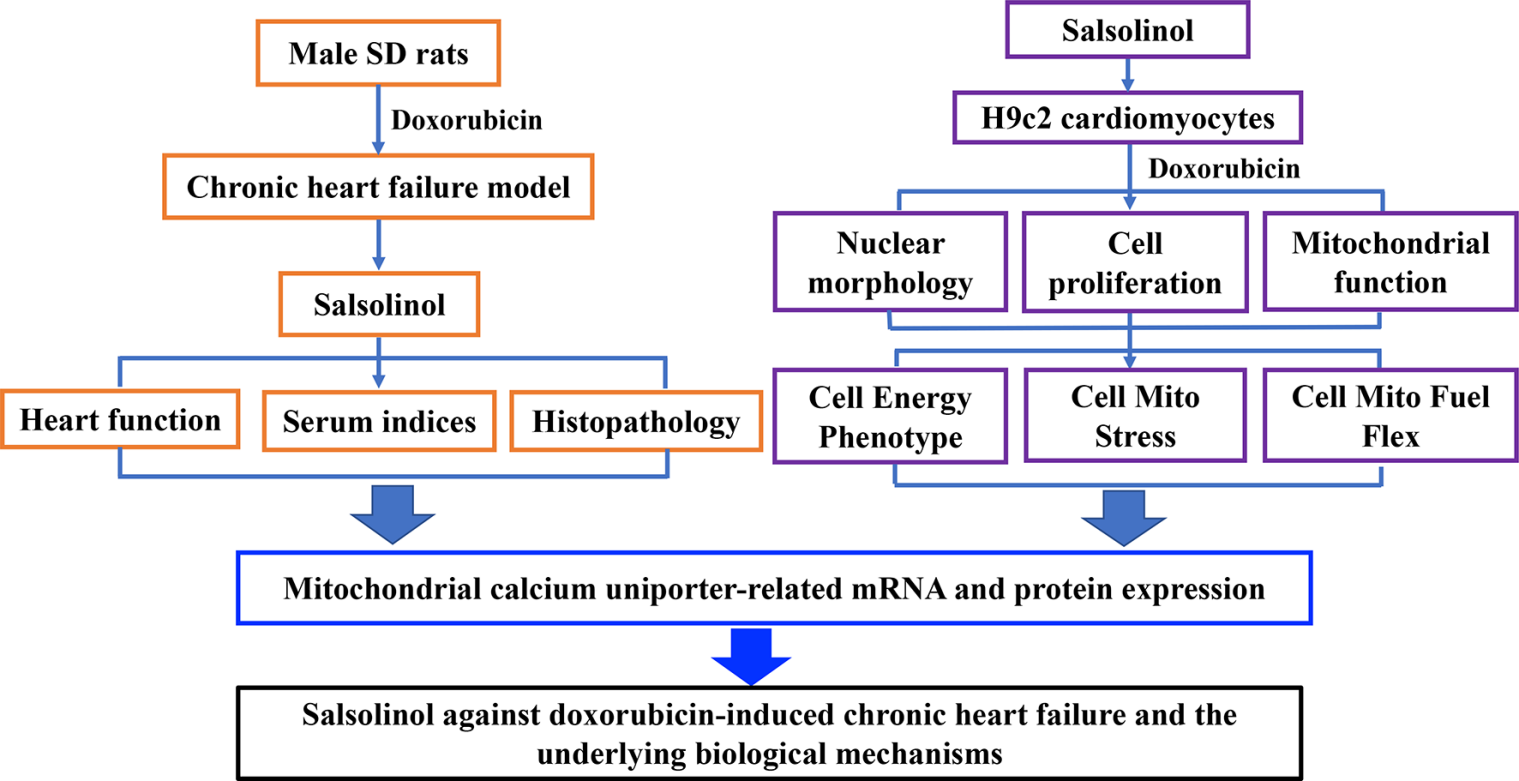
Research framework of this study. SD, Sprague-Dawley.

In this study, rats were intraperitoneally injected with DOX to establish CHF model. Therapeutic effects of SAL on hemodynamic parameters, serum indices, and the histopathology of the cardiac were analyzed *in vivo*. Moreover, the effect of SAL on cell viability, cardiomyocyte morphology, and mitochondrial membrane potential (MMP) of H9c2 cardiomyocytes were investigated by high-content screening (HCS) assay. Mitochondrial respiratory function and energy metabolism of H9c2 cardiomyocyte were measured by Seahorse Extracellular Flux (XFp) analyzer. To further investigate the potential mechanism of SAL, relative mRNA, and protein expression of key enzymes in the tricarboxylic acid cycle (TAC) *in vivo* were detected. Ultimately, mRNA and proteins expressions associated with the mitochondrial energy metabolism of H9c2 cells were explored. The results indicated that the therapeutic effects of SAL on CHF are possibly related to ameliorating cardiomyocyte function resulting in promotion of mitochondrial respiratory and energy metabolism. Furthermore, the potential mechanism might be related to downregulating MCU pathway. These findings may provide a potential therapy for CHF. The research process of this study is shown in **Figure 1**.

## Materials and Methods

### Materials

Standards of DOX (purity ≥98%, Cat. No. CHB160921) and SAL (purity ≥98%, Cat. No. CHB160922) were obtained from Chroma Biotechnology Co. Ltd (Chengdu, China). Ruthenium red (CAS No. 11103-72-3) and spermine (purity ≥96%, CAS No. 71-44-3) were purchased from Sigma Aldrich. All drugs above were dissolved in dimethyl sulfoxide (DMSO) and then diluted to corresponding concentration when used. DOX hydrochloride for injection (Shenzhen, China, batch number: 1809E2) was purchased from Shenzhen Main Luck pharmaceutical Inc. Dobutamine hydrochloride (DH) injection (Shanghai, China, batch number: 1803203) was obtained from SPH NO.1 Biochemical & Pharmaceutical Co., Ltd.

### Animal Handling

The experimental protocols were approved by the Ethics Committee of the Ethics of Animal Experiments of the Fifth Medical Center of PLA General Hospital (Approval ID: IACUC-2018-010). The present study was conducted according to the recommendations of the Guidelines for the Care and Use of Laboratory Animals of the Ministry of Science and Technology of China.

Male Sprague-Dawley rats (200 ± 20 g, *n* = 50) were obtained from Beijing Keyu Animal Breeding Center [Permission No. SYXK (Jing) 2018-0036]. As in our previous study (Wen et al., 2019), rats in the control groups (*n* = 12) were intraperitoneally injected with saline and in the model groups (*n* = 38) were intraperitoneally injected with DOX hydrochloride for injection to establish CHF model. The injection was not stopped until the accumulative doses of DOX were 15 mg/kg body weight (2.5 mg/kg body weight, twice a week for six times), which is a dose based on previous experiments (Siveski-Iliskovic et al., 1994; Bai et al., 2017). Notably, parameters, including left ventricular systolic pressure (LVSP), left ventricle end diastolic pressure (LVEDP), +left ventricle dp/dt_max_ (+LV dp/dt_max_), and −left ventricle dp/dt_max_ (−LV dp/dt_max_) in the control and model groups (three rats in each group) (Lu et al., 2017; Zhang et al., 2017), were comprehensively assessed using an RM6240 (Chengdu Instrument Factory, Sichuan, China) from the left ventricle *via* manometer after the last intraperitoneal treatment of DOX. The CHF model was successfully established when the values of +dp/dt_max_ decreased to less than 50% of the values of the control group.

The animals with successfully prepared CHF model were randomly divided into four different groups of eight rats in each, including the DOX group, DH (50 μg/kg), SAL low-dose group (5 mg/kg), and SAL high-dose group (10 mg/kg). The rats serving as a control and DOX group received an equal volume of normal saline. The other groups were intraperitoneally injected with corresponding drugs once a day for seven consecutive days. After the last treatment, heart function was assessed using a multi-channel physiological signal acquisition system. Finally, all the rats were sacrificed. The serum and heart tissue sample of each rats was collected and stored at −80°C for determining a series of indicators. Serum levels of brain natriuretic peptide (BNP), lactate dehydrogenase (LDH), renin, angiotensin (Ang)-II, and aldosterone (ALD) were measured on a Synergy H1 Hybrid Reader (Biotech, USA). The heart tissue was excised and fixed in 4% paraformaldehyde general tissue fixative. To detect apoptosis of myocardial cells, the paraffin-fixed heart sections were stained with the terminal deoxynucleotidyl transferase (TdT)-mediated dUTP nick-end labeling (TUNEL) technique.

### Cell Culture and Cell Viability Assay

The H9c2 rat cardiomyocyte cell line was obtained from the Cell Resource Center, IBMS, CAMS/PUMC (CRC/PUMC, Beijing, China). Cells were cultivated with Dulbecco’s modified Eagle’s medium (DMEM) supplemented with 10% fetal bovine serum (FBS) and 1% penicillin/streptomycin in a humidified incubator containing 5% CO_2_ at 37°C. Cell viability was detected using cell counting kit-8 (CCK-8; Cat. No. HY-K0301, MedChemExpress, USA). The optical density (OD) value was measured at 450 nm using a Synergy H1 Hybrid Reader (Biotech, USA).

### High-Content Analysis Experiments

The Array Scan High-Content System (Thermo Scientific, Massachusetts, USA) was used to detect nuclear morphology, cell proliferation, and mitochondrial function of H9c2 cells. Fluorescent dyes, including Hoechst 33342 (H3570, Invitrogen), calcein AM (C3099, Invitrogen), and ethidium homodimer-1 (EthD-1) (L3224, Invitrogen) were used to confirm the position and cell morphology and make quantitative analysis of H9c2 cells. Tetramethylrhodamine, ethyl ester, perchlorate (TMRE, T669, Invitrogen), a ∆ψ_m_-dependent cationic dye, was used to monitor MMP. Cell health profiling assay module was selected in the HCS system, and several different wavelength channels were set to collect fluorescence images. The measured parameters and format are similar to those used previously (Chen et al., 2014). An Array Scan XTI (The Array Scan software algorithm was used to perform analysis) was used to quantify the mean fluorescence intensity of H9c2 cardiomyocytes.

### Analysis of Mitochondrial Respiratory Function and Mitochondrial Energy Metabolism

**Figure 2:**
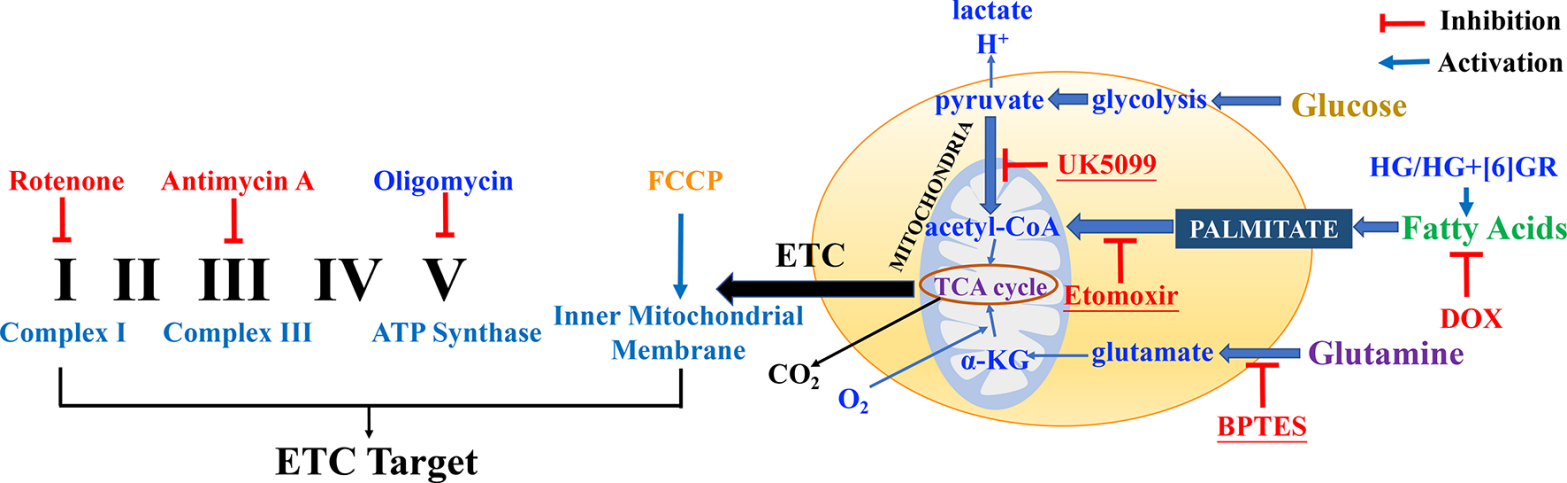
The complexes of the electron transport chain (ETC) and action targets of compounds in the mitochondrial respiratory function, energy metabolism, and fuel oxidation.

Mitochondrial respiratory function and mitochondrial energy metabolism were performed according to the manufacturer’s instructions. Briefly, H9c2 cells were plated at a density of 6 × 10^3^ cells per well into 8-well plates and incubated for 24 h. Then, H9c2 cells were pretreated with SAL (20 μM) followed by 5 μM of DOX and routinely incubated for 24 h. Prior to the assay, DMEM was replaced by Agilent Seahorse XF Base Medium containing 1 mM of pyruvate, 2 mM of glutamine, and 10 mM of glucose (adjusted pH to 7.4 with 0.1 N NaOH). Then, the compounds were loaded into the appropriate ports of a hydrated sensor cartridge. Cellular oxygen consumption rate (OCR), extracellular acidification rate (ECAR), and different indices were determined using the Seahorse XFp analyzer (XFp, Seahorse Biosciences, MA) according to the different manufacturers’ instructions. Among them, assay concentrations in the compound Seahorse XFp Cell Energy Phenotype Test were 10 μM of oligomycin and 10 μM of carbonyl cyanide-4-(trifluoromethoxy)phenylhydrazone (FCCP); the compounds in Seahorse XFp Cell Mito Stress Test were 10 μM of oligomycin, 10 μM of FCCP, and 5 μM of rotenone/antimycin; the compounds in Seahorse XFp Mito Fuel Flex Test were 4 μM of etomoxir, 3 μM of BPTES, and 2 μM of UK5099. Other relevant indicators were calculated according to the provided protocol. Cell counting was used for normalizing Seahorse XFp metabolic data to cellular parameters. H9c2 cells in each well were plated on cell counting slides, the dual chamber for cell counter (Bio-Rad, Cat. No. 145-0011), and counted using a TC10 automated cell counter (Bio-Rad, Singapore). Then, cell counting for normalization data was added to the normalized view for each assay result file using Wave 2.6.0 (Agilent Technologies, USA) before export. The normalization unit of the present study was 6 × 10^3^ cells. Data were analyzed using Seahorse XF Cell Test Report data analysis. The complexes of the electron transport chain (ETC) and action targets of compounds in mitochondrial respiratory function, energy metabolism, and fuel oxidation are shown in **Figure 2**.

### Reverse Transcription–Polymerase Chain Reaction (RT-PCR) Analysis *In Vivo* and *In Vitro*

Total mRNA was extracted from heart tissue and H9c2 cells using TRIzol reagent (Nordic Bioscience, Beijing, China) according to the manufacturer’s protocol. Quantitative real-time PCR (RT-PCR) for pyruvate dehydrogenase (PDH), malate dehydrogenase (MDH), and nicotinamide nucleotide transhydrogenase (NNT) mRNA in rats, MCU, MICU1, and MICU2 mRNA in H9c2 cardiomyocytes was performed and analyzed using cDNA and SYBR Green PCR Master Mix (Nordic Bioscience, Beijing, China). Primers sequences are listed in **Table 1**. The relative amounts of mRNA were determined based on 2^−∆∆Ct^ calculations with β-actin as the endogenous reference.

**Table 1:** Primers sequences used for real-time PCR analyses.

Gene	Forward (5′–3′)	Reverse (5′–3′)	prodSize
*PDH*	ACGAAGAGGAGGCTGTGCTAAGG	CGATACCGTTGCCGCCATAGAAG	80
*MDH*	CTGCTGTCATCAAGGCTCGGAAG	GGACACCATAGGAGTTGCCATCAG	147
*NNT*	GTTGCCTTGTCTCCTGCTGGTG	GCTTCGCCTGCTCCTGATTCC	80
*MCU*	ACGCTGCCAGTTCACACTCAAG	AAGTCATCGAGGAGCAGGAGGTC	162
*MICU1*	GACTGATGTTGGTGGCGTTCCTC	GTGGAGATTCTGCGTGAGCCTTC	87
*MICU2*	TGAGGCTGGCGGAGTTCAAGAG	CACACTCTGCTGCTGCGACAC	188
*β-Actin*	CACTATCGGCAATGAGCGGTTCC	CAGCACTGTGTTGGCATAGAGGTC	154

PCR, polymerase chain reaction.

### Western Blot Analysis to Detect the Protein Expression in H9c2 Cardiomyocytes

Total protein was extracted from the H9c2 cardiomyocytes using ice-cold radioimmunoprecipitation assay (RIPA) buffer supplemented with phenylmethylsulfonyl fluoride (PMSF). Protein concentration was determined using a bicinchoninic acid assay (BCA) protein assay kit (Lot. No. 20190215, Solarbio, Beijing, China) according to the manufacturer’s instructions. The polyvinylidene difluoride (PVDF) membranes were incubated with the primary antibodies, including rabbit anti-MCU antibody (D2Z3B, Cell Signaling Technology, dilution: 1:1,000), rabbit anti-CBARA1/MICU1 antibody (D4P8Q, Cell Signaling Technology, dilution: 1:1,000), rabbit anti-MICU2 antibody (A12198, ABclonal, dilution: 1:1,000), and anti-beta actin monoclonal antibody (ab115777, Abcam, dilution: 1:200). The antigen–antibody bands were detected using enhanced chemiluminescence (ECL) solution and visualized using X-ray film (Beyotime Institute of Biotechnology). Quantification of bands was performed by densitometric analysis using Bio-Rad Quantity One. β-Actin served as an internal control.

### Statistical Analysis

All data were presented as mean ± standard deviation (SD) and analyzed with the SPSS software program (version 19.0; SPSS Inc., Chicago, IL, USA). Data were presented using one-way ANOVA followed by *t*-test. The differences were considered to be statistically significant when *P* < 0.05 and highly significant when *P* < 0.01.

## Results

### DOX-Induced CHF in Rats in Terms of Cardiac Function

To identify whether CHF model had been successfully established, the role of DOX in rats was examined by hemodynamic parameters of cardiac function, including LVSP, LVEDP, and ± dp/dt_max_. Hemodynamics results showed that administration of DOX decreased LVSP and ± dp/dt_max_ and increased LVEDP in rats in comparison with the control group (*P* < 0.01, *P* < 0.01, *P* < 0.01) (**Table 2**). The results indicated the dysfunction of left ventricle systolic and diastolic. To this point, the CHF model in rats had been successfully prepared.

**Table 2:** DOX-induced myocardial injury in rats.

Group	n	LVSP (mmHg)	LVEDP (mmHg)	+dp/dt_max_ (mmHg/s)	−dp/dt_max_ (mmHg/s)
Control	3	121.65 ± 8.90	7.68 ± 0.94	5,865.51 ± 768.36	−5,040.13 ± 409.85
DOX	3	74.69 ± 8.80^**^	56.14 ± 8.11^**^	1,729.74 ± 158.23^**^	−1,524.40 ± 210.73^**^

Data are presented as mean ± SD; n = 3 per group. **P < 0.01 versus control group. DOX, doxorubicin; LVSP, left ventricular systolic pressure; LVEDP, left ventricle end diastolic pressure.

### Role of SAL on Cardiac Function

**Figure 3:**
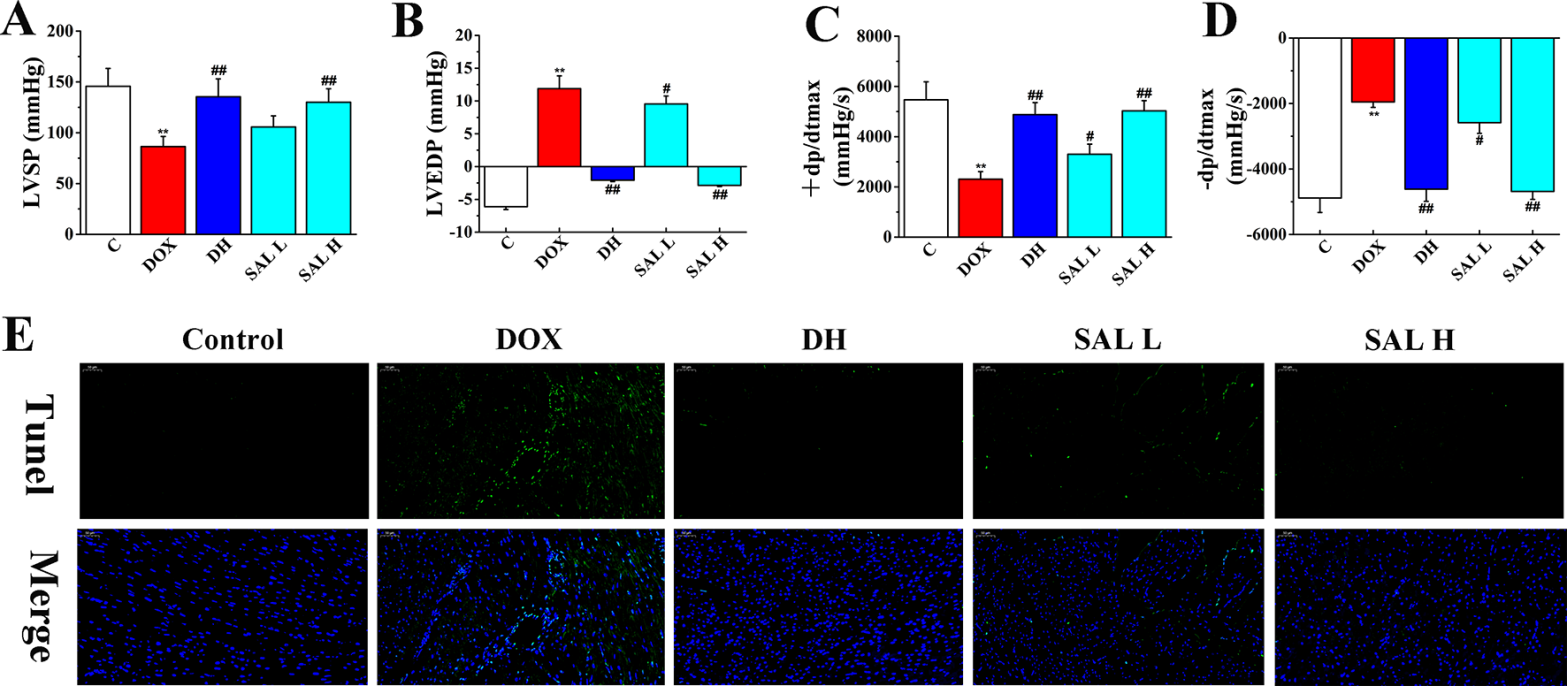
Therapeutic effect of SAL on DOX-induced CHF in rats. **(A)** LVSP, **(B)** LVEDP, **(C)** +LV dp/dt_max_, and **(D)** −LV dp/dt_max_ were determined by multi-channel physiological signal acquisition and processing system. **(E)** TUNEL stain of heart tissue. ^**^ *P* < 0.01 versus control group; ^#^ *P* < 0.05, ^##^ *P* < 0.01 versus DOX group. Data are presented as mean ± SD (*n* = 6). SAL, salsolinol; DOX, doxorubicin; CHF, chronic heart failure; LVSP, left ventricular systolic pressure; LVEDP, left ventricle end diastolic pressure.

Next, the effects of SAL on DOX-induced CHF were evaluated by measurements of cardiac function, including LVSP, LVEDP, and ± dp/dt_max_. As shown in **Figure 3**, the hemodynamic parameters of LVSP and ± dp/dt_max_ were markedly decreased in DOX-treated rats compared with the control group (*P* < 0.01, *P* < 0.01), which indicated left ventricle systolic and diastolic dysfunction. However, DH efficiently increased the hemodynamic parameters of LVSP and ± dp/dt_max_ and decreased LVEDP (*P* < 0.01, *P* < 0.01, *P* < 0.01). Similarly, the levels of LVSP and ± dp/dt_max_ in rats with 5 and 10 mg/kg of SAL significantly increased, and 10 mg/kg of SAL was almost equal to that of DH, indicating the enhancement effect of SAL on heart function.

To have sufficient evidence to show cardiac cell damage and its recovery, TUNEL staining was used to examine the cytotoxicity and visually present the effect of SAL on cardiac apoptosis induced by DOX. As shown in **Figure 3E**, the ratio of TUNEL positive myocardial cells was significantly increased in DOX-treatment group, indicating vast apoptosis of myocardial cytotoxicity and the ratio of TUNEL positive myocardial cells. Contrastively, SAL had an effect on alleviating the cardiomyocyte from apoptosis especially in SAL high-dose group, which indicated the anti-apoptotic activity of SAL. These results indicated that 10 mg/kg of SAL continuously and significantly protected heart tissues from CHF.

### Therapeutic Effects of SAL on DOX-Induced CHF Rats

As shown in **Table 3**, rats given DOX displayed remarkable increases in the BNP and LDH levels (*P* < 0.01, *P* < 0.01). Conversely, the serum levels of these two indexes were significantly reduced when rats were treated with DH. The effect of 10 mg/kg of SAL was almost equal to that of DH. In addition, the level of LDH but not that of BNP was also altered by 5 mg/kg of SAL (*P* < 0.05, *P* > 0.05). In addition, the serum levels of renin, Ang-II, and ALD were markedly enhanced in DOX-treated rats compared with the control group (*P* < 0.01, *P* < 0.01, *P* < 0.01). DH (50 μg/kg) efficiently decreased the serum levels of renin, Ang-II, and ALD. Similarly, the levels of renin, Ang-II, and ALD in rats with 5 and 10 mg/kg of SAL significantly decreased (*P* < 0.05, *P* < 0.01).

**Table 3:** Effects of SAL on serum BNP, LDH, renin, Ang-II, and ALD.

Group	n	BNP (ng/L)	LDH (ng/L)	Renin (pg/ml)	Ang-II (pg/ml)	ALD (pg/ml)
Control	6	55.66 ± 10.44	22.19 ± 5.29	57.77 ± 2.52	538.00 ± 52.84	118.79 ± 15.35
DOX	6	122.50 ± 18.98^**^	43.38 ± 6.01^**^	143.32 ± 17.15^**^	1,056.33 ± 94.49^**^	196.64 ± 33.29^**^
DH	6	63.81 ± 10.30^##^	28.49 ± 3.83^##^	66.14 ± 3.39^##^	615.50 ± 131.75^##^	128.99 ± 18.97^##^
SAL 5 mg/kg	6	113.99 ± 17.46	39.22 ± 5.69^#^	127.45 ± 14.28^##^	943.83 ± 100.00^#^	172.96 ± 15.63^#^
SAL 10 mg/kg	6	71.13 ± 11.79^##^	35.11 ± 4.16^##^	74.39 ± 9.03^##^	721.33 ± 130.66^##^	154.21 ± 32.61^##^

Data are expressed as mean ± SD. **P < 0.01 compared with control group; ^#^P < 0.05, ^##^P < 0.01 compared with DOX group. SAL, salsolinol; BNP, brain natriuretic peptide; LDH, lactate dehydrogenase; Ang-II, angiotensin-II; ALD, aldosterone; DOX, doxorubicin; DH, dobutamine hydrochloride.

### Relative mRNA Expression of Key Enzymes in the TAC

**Figure 4:**
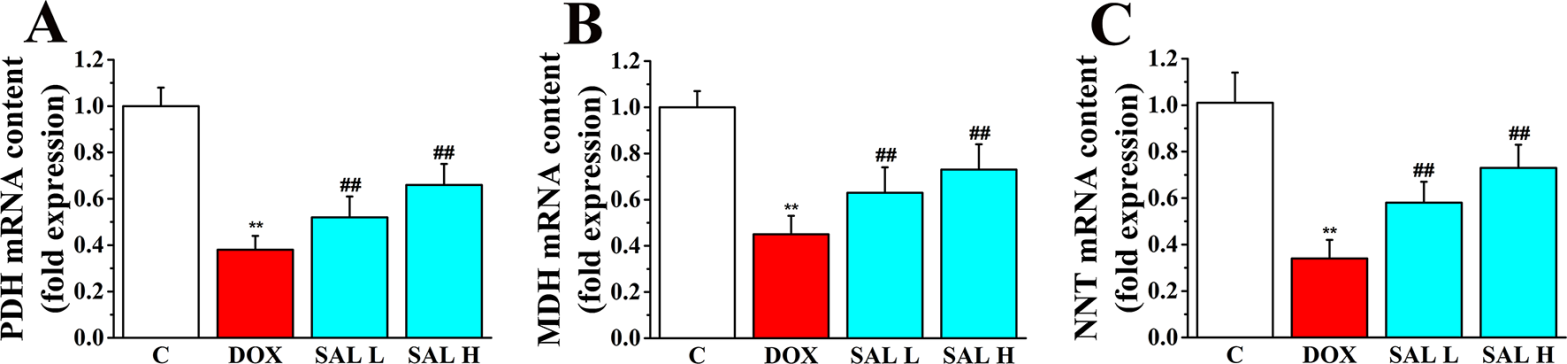
Effects of SAL on the key enzymes of the TAC. Rats were treated with different doses of SAL. The following three enzymes in the cardiac tissue were assayed: **(A)** PDH, **(B)** MDH, and **(C)** NNT (*n* = 6 per group). ^**^ *P* < 0.01 versus control group; ^##^ *P* < 0.01 versus DOX group. All data are presented as mean ± SD. SAL, salsolinol; TAC, tricarboxylic acid cycle; PDH, pyruvate dehydrogenase; MDH, malate dehydrogenase; NNT, nicotinamide nucleotide transhydrogenase; DOX, doxorubicin.

Research has shown that stimulation of the activity of key enzymes in TAC, including PDH, MDH, and NNT, leads to the promotion of regeneration of NADH and NADPH, enhances ATP synthesis in mitochondrial energy metabolism, and maintains the stability of mitochondrial environment (Zhang et al., 2017; Wen et al., 2019). Thus, the three key enzymes in the cardiac tissue were assessed by RT-PCR to evaluate the potential mechanism of SAL on the mitochondrial energy metabolism. As shown in **Figure 4**, the relative mRNA levels of PDH, MDH, and NNT decreased significantly in DOX group (*P* < 0.01, *P* < 0.01, and *P* < 0.01, respectively) (**Figures 4A–C**). Conversely, SAL could significantly increase the mRNA levels of PDH, MDH, and NNT in the cardiac tissue (*P* < 0.01, *P* < 0.01, and *P* < 0.01, respectively).

### SAL Suppressed DOX-Induced Cardiomyocyte Death in Cultured H9c2 Cells

**Figure 5:**
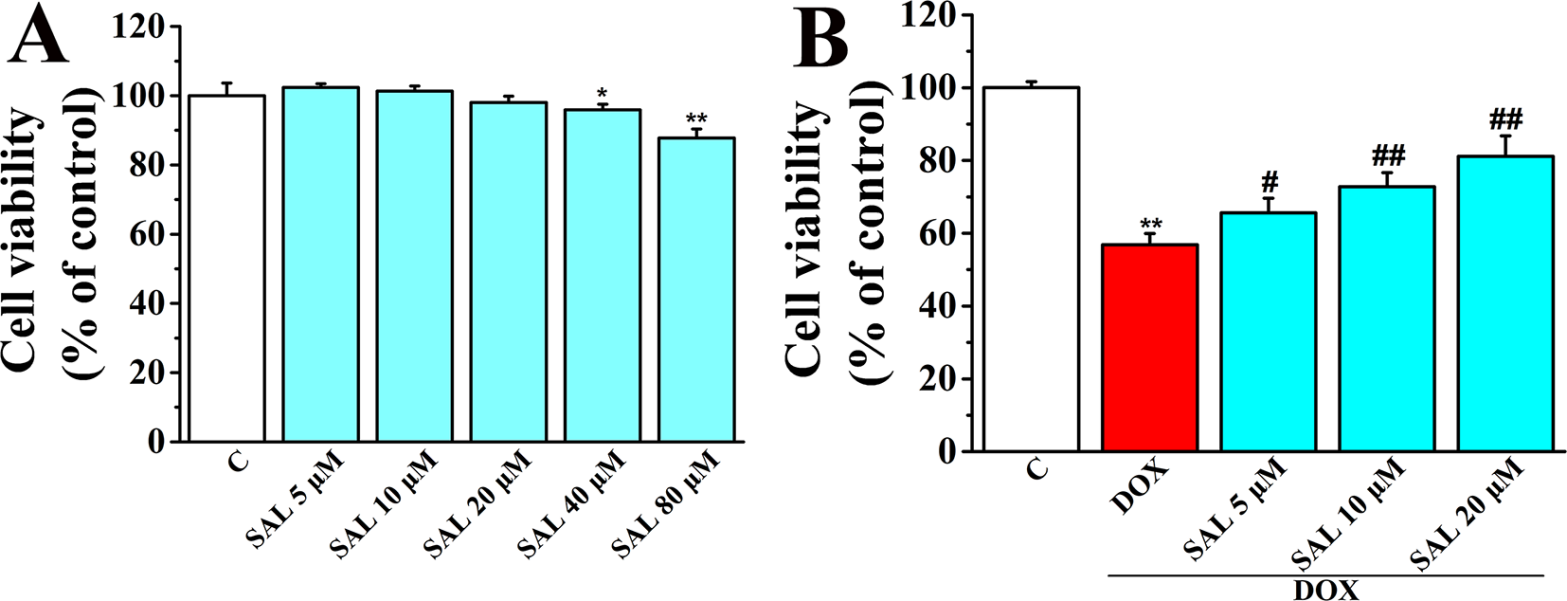
Effect of SAL on cell viability of H9c2 cells in the absence and presence of DOX. **(A)** Cell viability of H9c2 cells in the absence of DOX. **(B)** Cell viability of H9c2 cells in the presence of DOX. ^*^ *P* < 0.05, ^**^ *P* < 0.01 versus control group, ^#^ *P* < 0.05, ^##^ *P* < 0.01 versus DOX group (*n* = 6). Results were expressed as percentages of the control group. Data are shown as mean ± SD (*n* = 6). SAL, salsolinol; DOX, doxorubicin.

Cell viability of H9c2 cardiomyocytes was detected using CCK-8 kit to determine the optimum concentrations of SAL for protecting H9c2 cells against DOX-induced cytotoxicity. The results showed that SAL treatment for 24 h potently suppressed cell viability in a concentration-dependent manner with the increased concentrations (0, 5, 10, 20, 40, and 80 μM), among which SAL in the concentrations of 40 and 80 μM could significantly inhibit the cell viability compared with the control groups (*P* < 0.05, *P* < 0.01) (**Figure 5A**). Thus, the optimal concentration was not more than 40 μM. Then, H9c2 cells were pretreated with different concentration of SAL (0, 5, 10, and 20 μM) for 2 h. Cell viability was significantly decreased to 56.85 ± 3.06% (*P* < 0.01) when treated with 5 μM of DOX, compared with the control group. Cell viability of H9c2 cells was dramatically increased to 65.59 ± 4.04%, 72.80 ± 3.82%, and 81.12 ± 5.64% (*P* < 0.05, *P* < 0.01, *P* < 0.01) (**Figure 5B**), respectively, when pretreated with 5, 10, and 20 μM SAL, compared with the DOX group. The results indicated that SAL could remarkably promote cell viability in a concentration-dependent manner in H9c2 cells, and 20 μM of SAL showed a relatively good protective effect. Therefore, 20 μM of SAL was used in this study to investigate the protective effect of SAL on H9c2 cardiomyocyte injury induced by DOX.

### Effect of SAL on Nuclear Morphology and Cell Proliferation of H9c2 Cells

**Figure 6:**
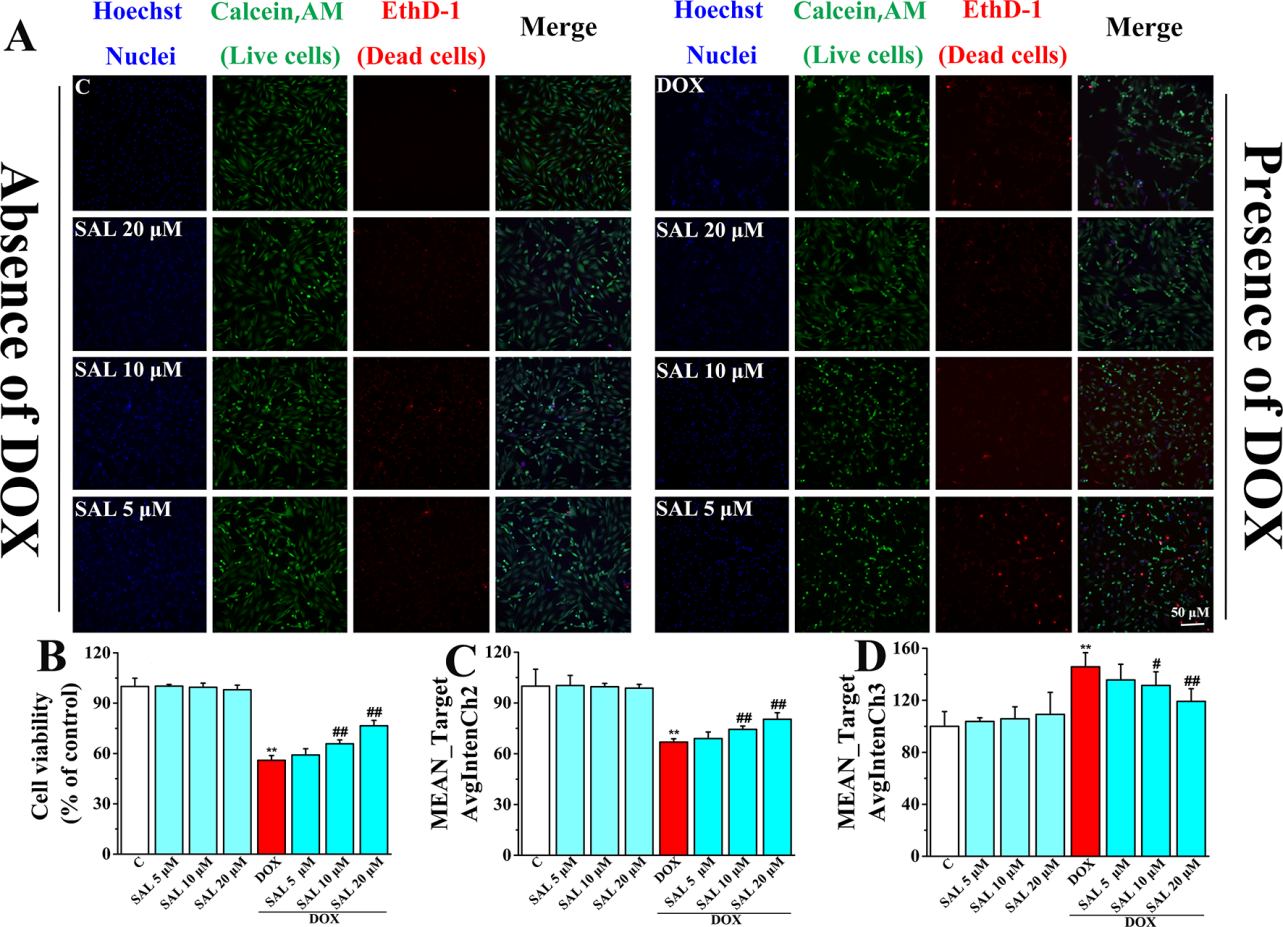
Representative photomicrographs and summary data for HCS image analysis of H9c2 cells. **(A)** Representative photomicrographs for HCS analysis of H9c2 cells; scale bar = 50 μm. **(B)** Valid cell count for HCS analysis of H9c2 cells (% of control). **(C)** Live cell count (MEAN_TargetAvgIntenCh2). **(D)** Dead cells count (MEAN_TargetAvgIntenCh3). ^*^ *P* < 0.05, ^**^ *P* < 0.01 versus control group, ^#^ *P* < 0.05, ^##^ *P* < 0.01 versus DOX group. Results are expressed as percentages of control group. Data are shown as mean ± SD (*n* = 6). HCS, high-content screening; SAL, salsolinol; DOX, doxorubicin; EthD-1, ethidium homodimer-1.

To more directly present the effect of SAL on cell morphological influence, high-content live-cell imaging assays were used to qualitatively and quantitatively assay cell count, morphology, and cell viability of H9c2 cells. Among them, nucleus staining (blue fluorescence), cell cytoplasm labeling (green fluorescence), and dead cells (red fluorescence) were marked by Hoechst 33342, calcein AM, and EthD-1, respectively. In the absence of the DOX group, including a control group and different concentrations of SAL, H9c2 cells possessed a homogenous Hoechst and calcein AM fluorescence in the nucleus and cytoplasm. However, there were alterations in morphological after treating cells with 5 μM of DOX, compared with the control group. Fortunately, SAL could mitigate H9c2 cells from cytotoxicity regarding morphological alterations, especially 20 μM (**Figure 6A**). As for cell count, it was significantly decreased after treated with DOX for 24 h (*P* < 0.01), compared with the control group. The reduction rate of cell number in the SAL group, compared with the model group, had significantly decreased (*P* < 0.01) (**Figure 6B**). In addition, the green fluorescence of the model group, compared with the control group, was significantly reduced (*P* < 0.01), and red fluorescence was significantly increased (*P* < 0.01), suggesting the decrease of live cells and increase of dead cells. SAL could certainly increase the green fluorescence and decrease the red fluorescence of H9c2 cells (*P* < 0.01) (**Figures 6C, D**). These results suggested that SAL could significantly ameliorate nuclear morphology and cell proliferation in DOX-induced cardiomyocyte injury and cytotoxicity in H9c2 cells.

### Effects of SAL on DOX-Induced MMP Reduction on H9c2 Cells

**Figure 7:**
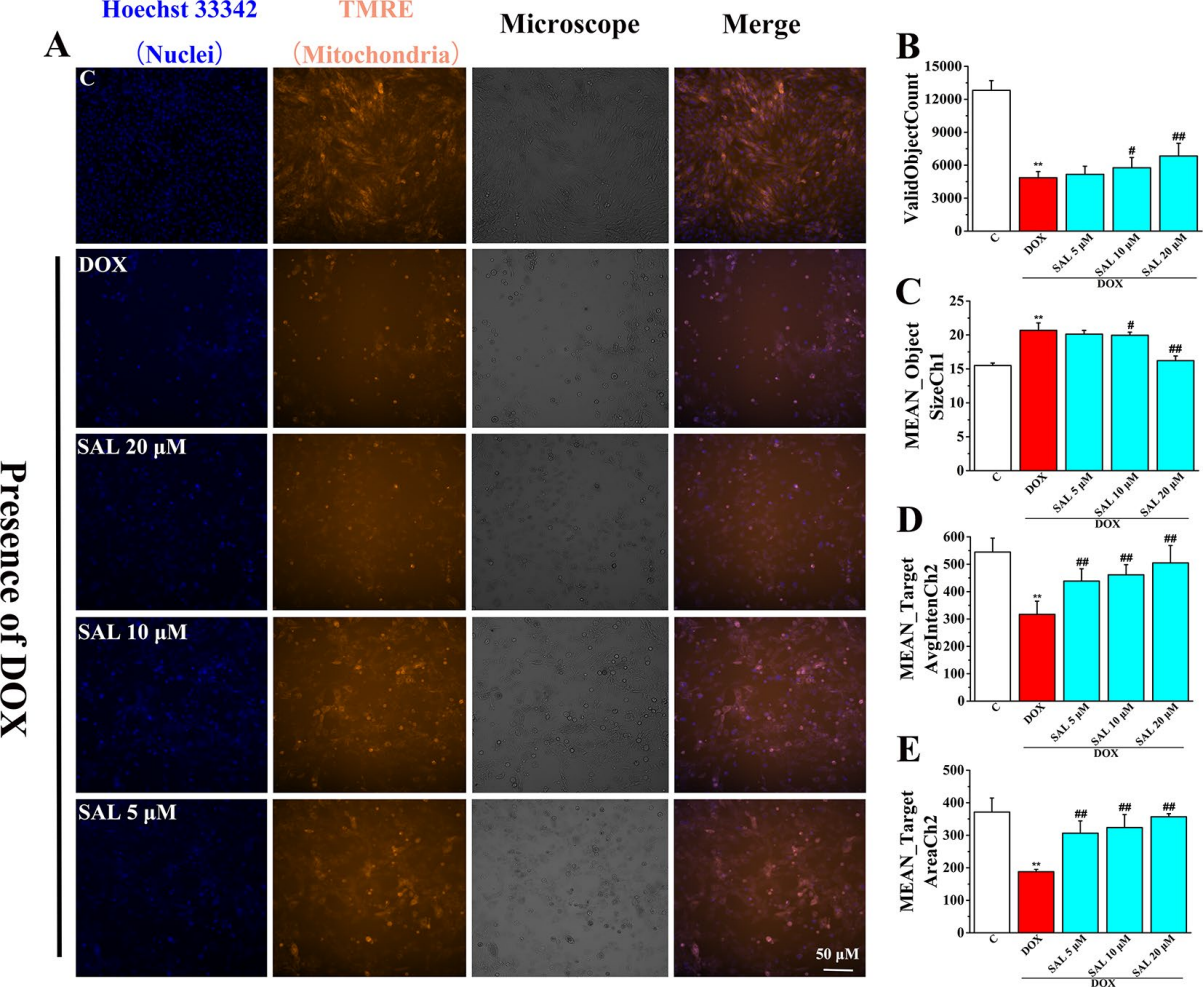
Representative images and corresponding numerical data for some typical cytotoxic effects of SAL on H9c2 cells nuclei and mitochondria. **(A)** Representative photomicrographs and summary data for TMRE staining of H9c2 cells. Scale bar = 50 μm. **(B)** Effect of different drugs on valid object cell count of H9c2 cells. **(C)** Effect of different drugs on nuclear size of H9c2 cells (MEAN_ObjectSizeCh1). **(D)** Effect of different drugs on average brightness of functional mitochondrial mass (A). **(E)** Effect of different drugs on projected area occupied by functioning mitochondrial mass (MEAN_TargetAreaCh2). ^**^ *P* < 0.01 versus control group, ^#^ *P* < 0.05, ^##^ *P* < 0.01 versus DOX group. All data are presented as mean ± SD (*n* = 6). SAL, salsolinol; DOX, doxorubicin.

To investigate the effects of SAL on mitochondrial functions of H9c2 cells, MMP was measured by HCS assay. As shown in **Figure 7**, fluorescent images of H9c2 cells stained with TMRE showed that cells emitted red–orange fluorescence in the control group, suggesting that MMP stayed in a normal state. The fluorescence intensity of TMRE decreased significantly in the DOX group, compared with the control group, suggesting that DOX caused the decrease of MMP (**Figure 7A**). That is, DOX may have caused the opening of mitochondrial permeability transition pore. Conversely, pretreatment with SAL could significantly enhance the intensity of the orange–red fluorescence decreased by DOX. Moreover, we also found that cells treated with SAL increased DOX-induced decrease in cell count (ValidObjectCount) (**Figure 7B**), average brightness of functional mitochondrial mass (Mean_TargetAvgIntenCh2) (**Figure 7D**), and the projected area occupied by functioning mitochondrial mass (Mean_TargetAreaCh2) (**Figure 7E**). Simultaneously, SAL decreased nuclear size (Mean_ObjectSizeCh1) than did the DOX group (**Figure 7C**). These results suggested that DOX caused the decrease of MMP and TMRE brightness but increased nuclear size, indicating mitochondrial depolarization or a mitochondrial structural change. Thus, SAL may reduce DOX-induced mitochondrial dysfunction by affecting MMP.

### SAL Promoted Mitochondrial Respiratory Function and Energy Metabolism

**Figure 8:**
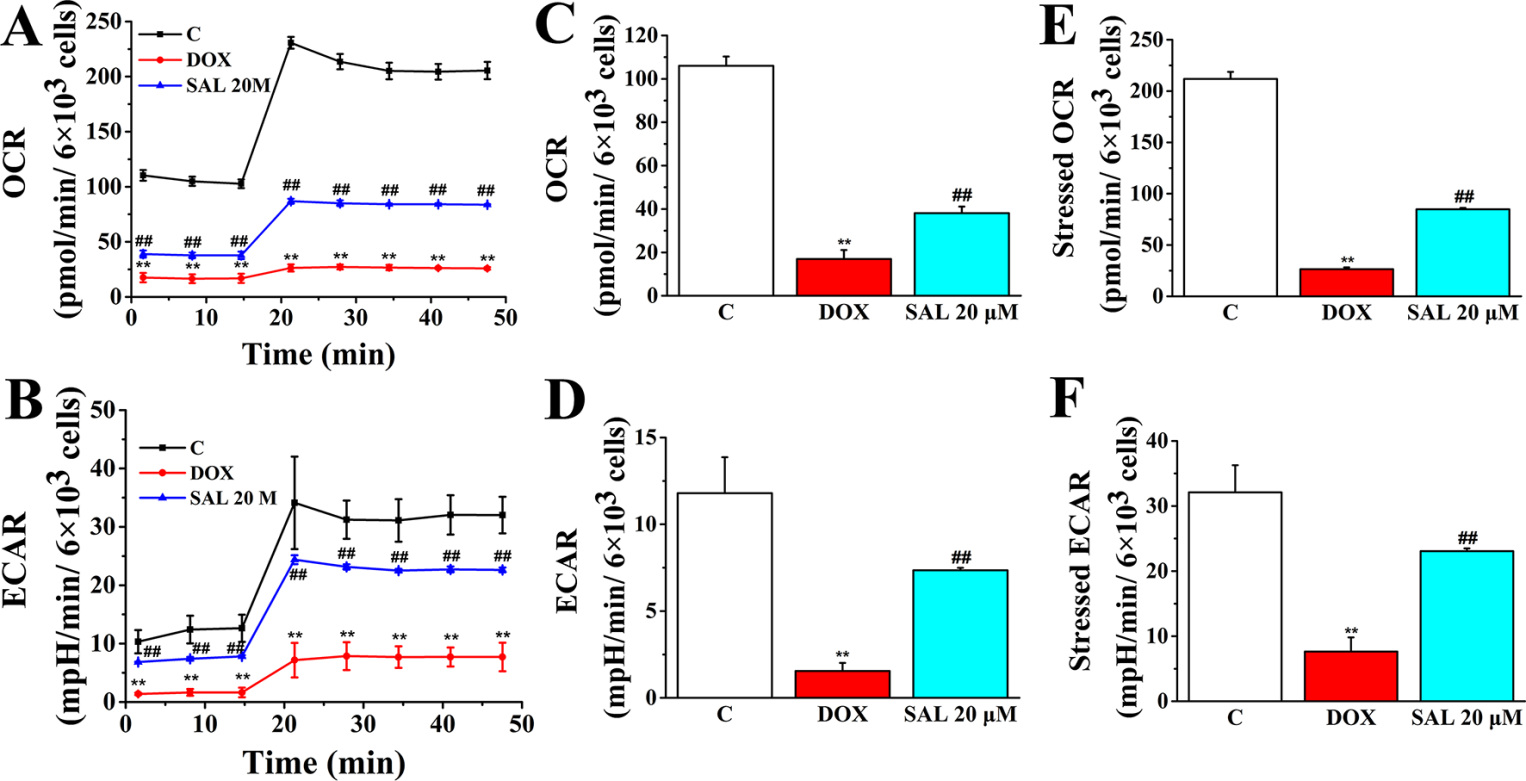
Pretreatment with SAL could attenuate the inhibition effect of DOX in mitochondrial respiration. Mitochondrial respiration was measured by a Seahorse XFp apparatus detecting the basal OCR and ECAR. **(A)** OCR. **(B)** ECAR. **(C)** Baseline OCR. **(D)** Baseline ECAR. **(E)** Stressed OCR. **(F)** Stressed ECAR. ^**^ *P* < 0.01, versus control group; ^##^ *P* < 0.01, versus DOX group. All data are presented as mean ± SD (*n* = 3). SAL, salsolinol; DOX, doxorubicin; OCR, oxygen consumption rate; ECAR, extracellular acidification rate.

To assess whether the observed toxicity of DOX was correlated with mitochondrial respiration function, Seahorse XFp analyzer was used to determine the value of OCR and ECAR in H9c2 cells. The results indicated that treatment with DOX for 24 h specifically impaired both OCR and ECAR (**Figures 8A, B**). Interestingly, baseline OCR (**Figure 8C**), baseline ECAR (**Figure 8D**), stressed OCR (**Figure 8E**), and stressed ECAR (**Figure 8F**) were significantly promoted in H9c2 cells when pretreated with 20 μM of SAL, compared with the DOX group, for 2 h (*P* < 0.01, *P* < 0.01, *P* < 0.01, and *P* < 0.01). These results demonstrated that DOX treatment causes mitochondrial dysfunction and that pretreatment of SAL could partially prevent these effects.

**Figure 9:**
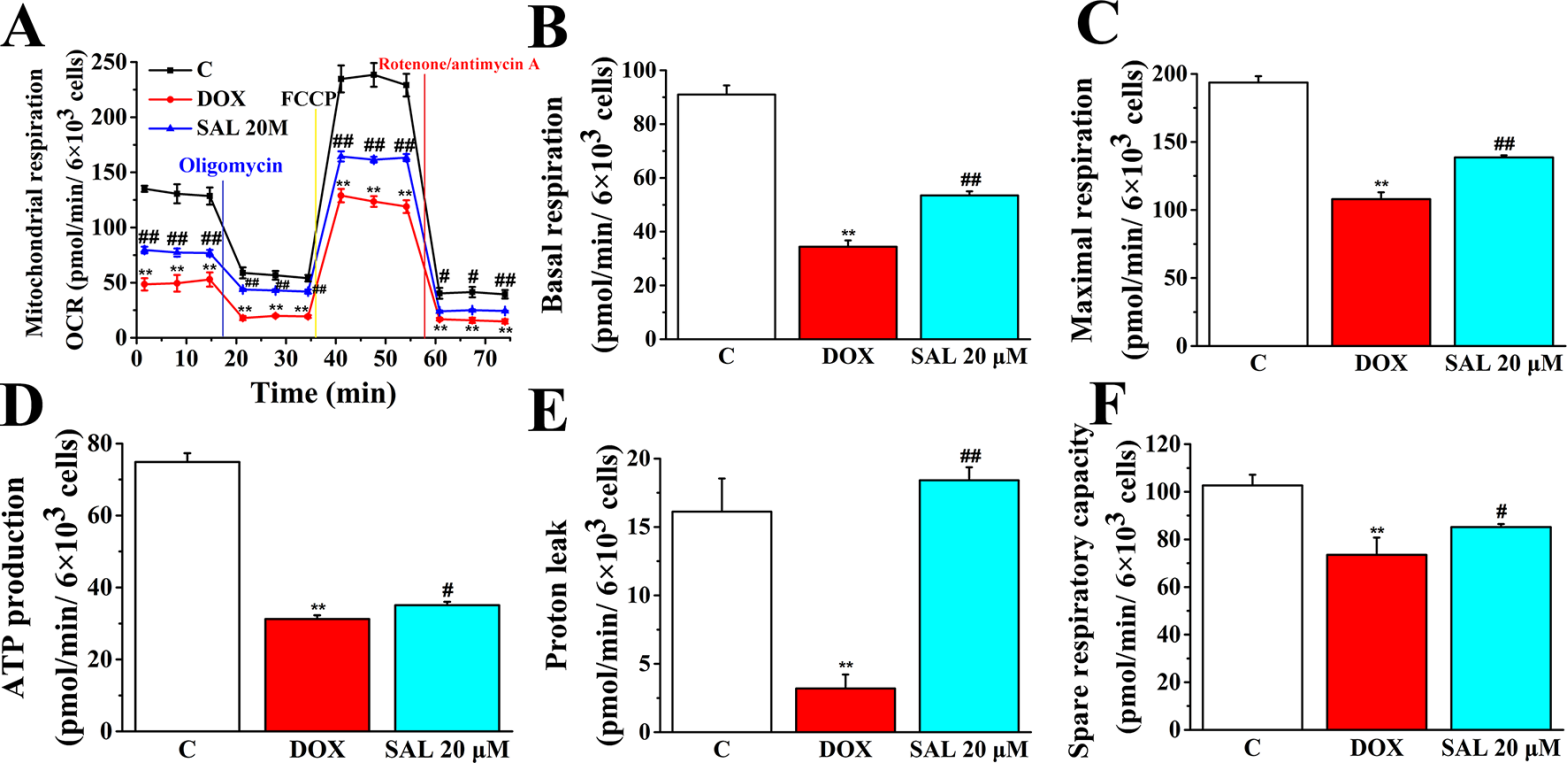
SAL rescues mitochondrial respiration and energy metabolism in DOX-treated H9c2 cells. During testing, H9c2 cells were treated with 10 μM of oligomycin, 10 μM of FCCP, and 5 μM of rotenone/antimycin A. **(A)** OCR, **(B)** basal respiration, **(C)** maximal respiration, **(D)** ATP production, **(E)** proton leak, and **(F)** spare respiratory capacity were assessed using a Seahorse Bioscience XFp analyzer. ^**^ *P* < 0.01 versus control group. ^#^ *P* < 0.05; ^##^ *P* < 0.01 versus DOX group. All data are presented as mean ± SD (*n* = 3). SAL, salsolinol; DOX, doxorubicin; FCCP, carbonyl cyanide-4-(trifluoromethoxy)phenylhydrazone; OCR, oxygen consumption rate; ECAR, extracellular acidification rate.

To further understand the mechanism responsible for the protective effects of SAL, mitochondrial respiratory function and energy metabolism in H9c2 cells were measured using the Agilent Seahorse XFp Cell Mito Stress Test. Cells were treated with SAL (20 μM) for 2 h before DOX treatment. As expected, mitochondrial oxidative phosphorylation, as measured by OCR, was reduced in the DOX group compared with the control group. Impressively, OCR was significantly higher in the SAL group (**Figure 9**). Simultaneously, we observed that DOX could significantly decrease the basal respiration (*P* < 0.01) (**Figure 9B**), maximal respiration (*P* < 0.01) (**Figure 9C**), ATP production (*P* < 0.01) (**Figure 9D**), proton leak levels (**Figure 9E**), and spare respiratory capacity (*P* < 0.01) (**Figure 9F**) in H9c2 cells compared with control group. Notably, SAL could partially prevent these effects and significantly elevate basal respiration (*P* < 0.01) (**Figure 9B**), maximal respiration (*P* < 0.01) (**Figure 9C**), and proton leak (**Figure 9E**) levels, which were inhibited by DOX. In addition, SAL could significantly enhance ATP production (*P* < 0.05) (**Figure 9D**). The effects of SAL on respiration were also reflected in the spare respiratory capacity of the mitochondria, as SAL increased spare oxidative capacity and attenuated the inhibition of spare capacity by DOX treatment (*P* < 0.05) (**Figure 9F**). These results support the finding that the potential protective effects of SAL against DOX-induced mitochondrial dysfunction in H9c2 cells may be related to the promotion of ATP production.

**Figure 10:**
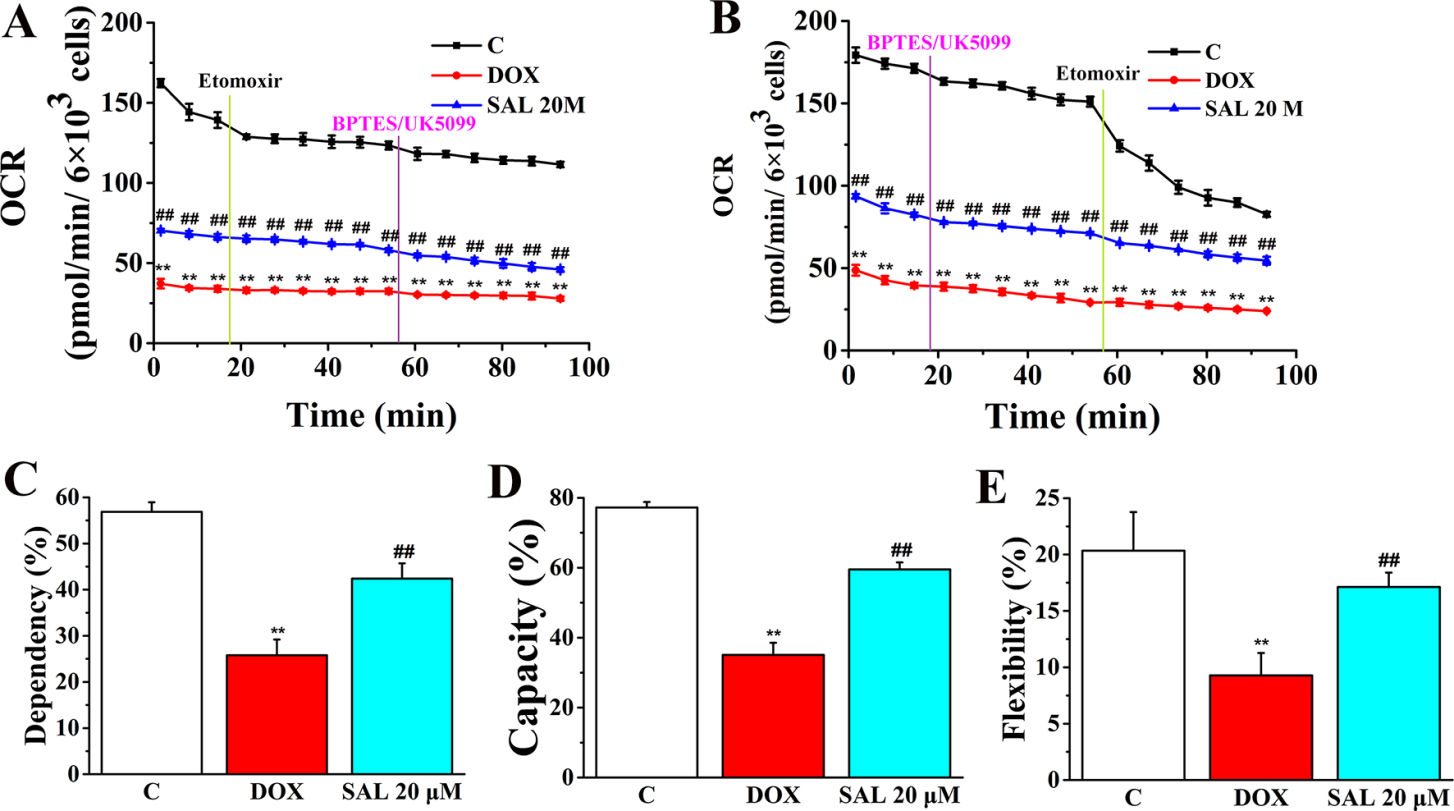
SAL ameliorates H9c2 cells from DOX-induced decrease on mitochondrial fuel flex. Mitochondrial substrate analysis was determined. During testing, H9c2 cells were treated with 4 μM of etomoxir and 3 μM of BPTES/2 μM of UK5099 in succession. **(A)** Effects of SAL on fuel dependency in terms of the fatty acid oxidation pathway. **(B)** Effects of SAL on fuel capacity in terms of fatty acid oxidation pathway and oxidation rates of fatty acids expressed in dependency **(C)**, capacity **(D)**, and flexibility **(E)** to maintain baseline OCR levels determined with the Seahorse XFp respirometer. ^**^ *P* < 0.01 versus control group. ^##^ *P* < 0.01 versus DOX group. All data are presented as mean ± SD (*n* = 3). SAL, salsolinol; DOX, doxorubicin; OCR, oxygen consumption rate.

The rate of oxidation of each fuel (glutamine, glucose, and fatty acids) was determined by measuring OCR of H9c2 cells in the presence or absence of corresponding fuel pathway inhibitors. By blocking fatty acid metabolism with etomoxir (carnitine palmitoyltransferase-I inhibitor of fatty acid mitochondrial import) and subsequent inhibiting the glucose and glutamine pathways with UK5099 and BPTES, respectively, we found that DOX-treated H9c2 cells, compared with control groups, have a decreased OCR and dependency on fatty acids, while SAL-treated groups have an increased OCR and dependency to maintain baseline respiration (**Figures 10A, C**). In addition, by first inhibiting the glucose and glutamine pathway followed by blocking fatty acid metabolism, H9c2 cells show an increased capacity of fatty acid utilization to meet energy demand when other fuel pathways are inhibited (**Figures 10B, D**). This indicates that compared with blocking fatty acid first, H9c2 cells are able to maintain higher levels of ATP when energy production is primarily fatty acid dependent. There are still significant decreases in terms of flexibility of fatty acid in the DOX group (*P* < 0.01). Significant increase differences are observed in the flexibility of fatty acid oxidation in the SAL group (*P* < 0.01) (**Figure 10E**).

### Relative Expression of MCU mRNA, MICU1 mRNA, and MICU2 mRNA in H9c2 Cells

**Figure 11:**
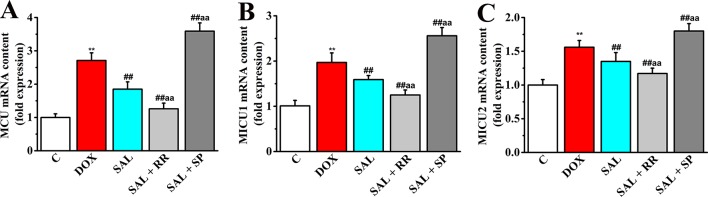
Effects of SAL on the mRNA expression levels of MCU, MICU1, and MICU2 in H9c2 cells. The mRNA expression levels of MCU **(A)**, MICU1 **(B)**, and MICU2 **(C)** were detected by RT-PCR in different groups. ^**^ *P* < 0.01 versus control group. ^##^ *P* < 0.01 versus DOX group. ^aa^ *P* < 0.01 versus SAL group. All data are presented as mean ± SD (*n* = 3). SAL, salsolinol; DOX, doxorubicin; MCU, mitochondrial calcium uniporter; MICU1, mitochondrial calcium uptake 1; MICU2, mitochondrial calcium uptake 2; RT-PCR, reverse transcription–polymerase chain reaction.

Given that MCU can transfer Ca^2+^ from cytoplasm to mitochondrial matrix and controls its rate by electrochemical gradient, which has great significance in intracellular Ca^2+^ signal transduction, Ca^2+^ homeostasis, and mitochondrial energy metabolism (O’Brien et al., 2006), we next asked whether SAL could promote mitochondrial energy metabolism of H9c2 cells *via* regulating mRNA expression of MCU and its regulatory mRNA and whether the pharmacological inhibition of MCU could affect the effects of SAL. To this end, MCU inhibitor ruthenium red and MCU activator spermine were pretreated, and the expression of metabolism-related genes including MCU, MICU1, and MICU2 were determined by RT-PCR. Although the gene types of energy metabolism varied somewhat, all conditions showed similar results that DOX increased expression of MCU and its regulatory mRNA to values in H9c2 cells (*P* < 0.01). Furthermore, pretreatment with the MCU inhibitor ruthenium red (10 μM) following SAL (20 μM) resulted in lower expression of MCU, MICU1, and MICU2, but pretreatment with the MCU activator spermine (20 μM) following SAL (20 μM) resulted in higher expression in these genes (**Figure 11**).

### Protein Expression of MCU, MICU1, and MICU2 in H9c2 Cells

**Figure 12:**
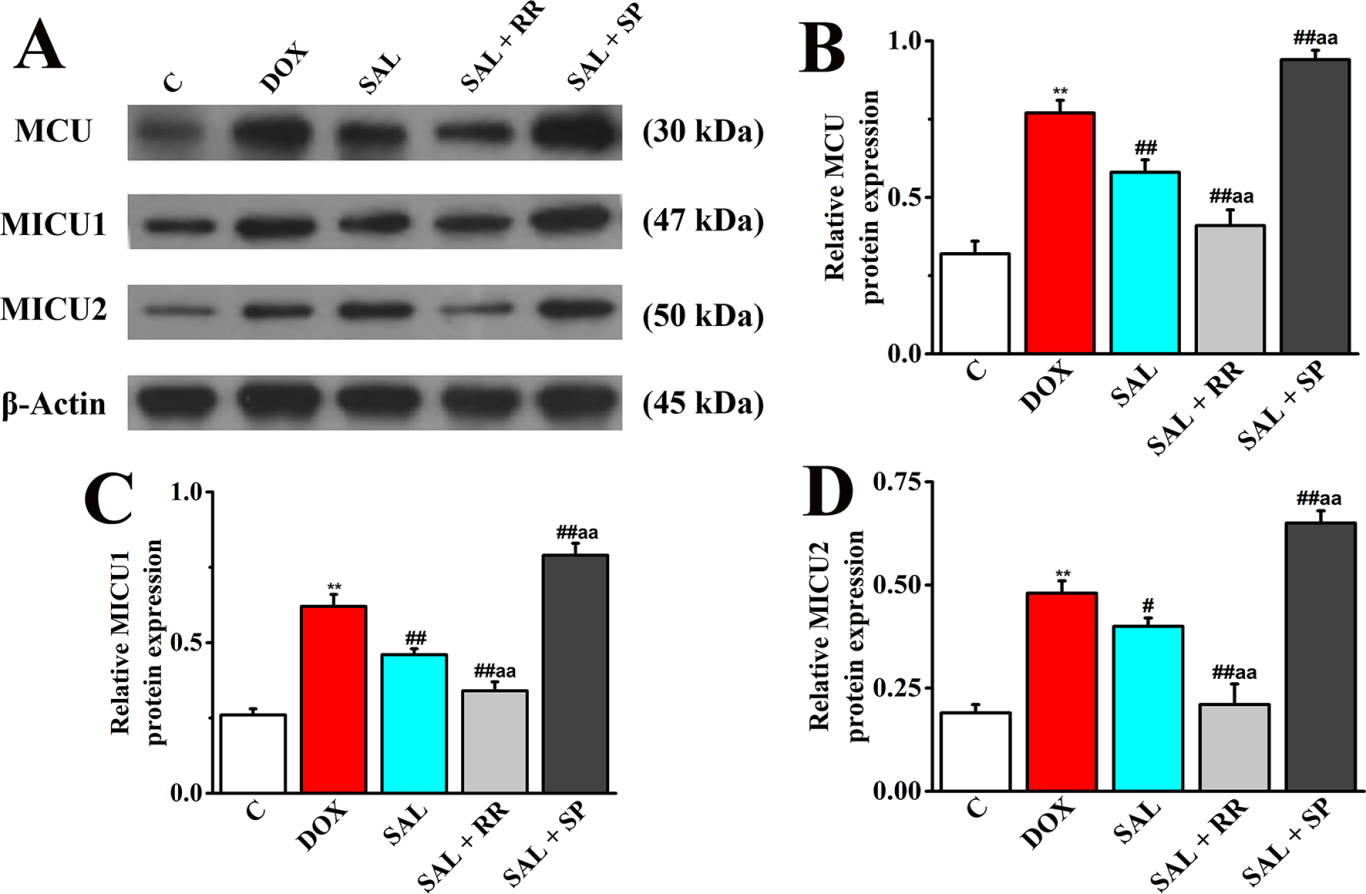
Effects of SAL on the protein expression levels of MCU, MICU1, and MICU2, in H9c2 cells. **(A)** The Western blot images of MCU, MICU1, and MICU2. **(B)** Relative MCU protein level in H9c2 cells. **(C)** Relative MICU1 protein level in H9c2 cells. **(D)** Relative MICU2 protein level in H9c2 cells. ^**^ *P* < 0.01 versus control group; ^#^ *P* < 0.05; ^##^ *P* < 0.01 versus DOX group. ^aa^ *P* < 0.01 versus SAL group. All data are presented as mean ± SD (*n* = 3). SAL, salsolinol; MCU, mitochondrial calcium uniporter; MICU1, mitochondrial calcium uptake 1; MICU2, mitochondrial calcium uptake 2.

To further confirm our speculation that MCU plays an important role in the energy metabolism-related signaling pathway in H9c2 cells, the protein expression of MCU and its regulatory protein, MICU1, and MICU2 in different groups with or without MCU inhibitors ruthenium red (10 μM) or MCU activator spermine (20 μM) were detected. The DOX group showed increased levels of MCU, MICU1 protein, and MICU2 protein compared with expression levels in the control group (**Figure 12**), consistent with the expression of mRNA. However, SAL treatment can significantly decrease these protein expressions. Conversely, the enhancement could be inhibited by MCU inhibitor ruthenium red. Interestingly, we found that in addition to MCU, the expressions of MICU1 and MICU2 were also decreased in the MCU inhibitors group but increased in the MCU activator spermine group. This phenomenon provided further evidence that SAL may relieve H9c2 cells from DOX-induced cardiomyocyte injury *via* the downregulation of MCU activity.

## Discussion

The present study had provided the pharmacodynamics and mechanism evidence that SAL treatment attenuated the DOX-induced cardiac dysfunction *in vivo*, as well as mitochondrial respiratory function injury and energy metabolism dysfunction *in vitro*. Based on the positive inotropic and energy metabolism effects, these results suggested that SAL may be the active components of *Fuzi* in treatment CHF. To the best of our knowledge, this is the first report describing how SAL protects against DOX-induced CHF *in vivo* and *in vitro*.

DOX, also known as adriamycin, is widely used as one of the most effective anti-cancer therapeutics known to suppress mitochondrial function leading to mitochondrial toxicity. Thus, the clinical application of DOX is limited by these irreversible and cumulative side effects (Baughman et al., 2011). Currently, excessive mechanisms have been proposed to account for DOX-induced cardiotoxicities, such as cardiac energy homeostasis, intracellular calcium disturbance, oxidative stress, dysregulation of metabolites, inflammation, and apoptosis of cardiomyocytes (Jones et al., 2004; Cui et al., 2017). In addition, DOX is known to inhibit mitochondrial function and induce mitochondrial energy metabolism disorder in cardiomyocytes (Guo et al., 2018), which can lead to a dose-dependent, cumulative, and permanent degenerative cardiomyopathy (Hakuno et al., 2013; Hosseini et al., 2017). Although the relationship among the events of DOX-induced cytotoxicity, mitochondrial respiratory function injury, and energy metabolism disorder is not well defined, which can induce injury in cardiomyocyte function *in vitro*. Thus, DOX was used to establish a cardiomyocyte mitochondrial metabolism injury model. In the present study, the ability of SAL to increase cell viability, mitochondrial OCR and ECAR, basal respiration and maximal respiration, and ATP production may contribute to the protective role of reducing H9c2 cardiomyocyte mitochondrial energy metabolism disorder and respiratory function injury from DOX injury. Research also explains drugs that not only promote mitochondrial energy metabolism but also partially prevent the cell from DOX-induced mitochondrial function, which will benefit a lot in terms of cell metabolism and capacity of ATP production.

ALRP is a potent traditional herbal medicine extensively used in the treatment of cardiovascular diseases in many Asian countries (Yang et al., 2018). For several decades, researchers had paid more attention to investigate the cardioactive components of ALRP and verified the definite cardiac effects of ALRP (Liu et al., 2012; Zhou and Liu, 2013). Regarding the bidirectional effect of ALRP on cardiotoxicity and cardioprotection, researchers had found that the toxic component of ALRP is diester-type diterpene ester-soluble alkaloids represented by aconitine, and the cardiovascular active substance is heat-resistant water-soluble components (Yang et al., 2018). Among the cardioprotective components, SAL, a product of nonenzymatic condensation of dopamine and acetaldehyde, produces a dose-dependent positive chronotropic effect on the rat isolated perfused heart (Liu et al., 2012). Thus, SAL plays an important role in regulating heart performance. In addition, it is valued for playing a role in the biosynthesis of catecholamines in the body (Sokolova et al., 1990). SAL can be produced in the body and inhibits the activity of catechol-*O*-methyltransferase, which itself methylates the free hydroxy group under the action of the enzyme, thereby changing the metabolic rate of catecholamines such as dopamine *in vivo* (Melchior, 1979). Experiments have shown that SAL is a weak beta-adrenergic stimulant that excites the guinea pig’s isolated atrium and increases the frequency of contraction and blood pressure in normal and ruined spinal cord rats and demonstrates excitatory effects on both β- and γα-adrenergic bodies (Melis et al., 2015). Although Chen and Liang (1982) isolated this compound from aconite in 1979, the cardiotonic effect of SAL is still not comprehensively evaluated and confirmed in current studies.

To investigate the therapeutic effects of SAL, Sprague-Dawley rats were intraperitoneally injected with DOX to induce CHF model. Then, hemodynamic indexes were performed to evaluate cardiac function in rats. Our results showed that the values of ± dp/dt_max_ in model groups were reduced to 50% those of the control group, indicating the CHF model was successfully prepared. Next, SAL was intraperitoneally administered to investigate whether it could produce a therapeutic effect on DOX-induced CHF. The results showed that compared with the DOX group, SAL could significantly improve heart function, reducing levels of myocardial injury markers and relieving cardiomyocyte apoptosis. Recent researches (Francesco et al., 2017; Ichiki et al., 2017; Vecchis and Ariano, 2018) have shown that RAAS plays a key role in CHF treatment. Therefore, serum levels of renin, Ang-II, and ALD were measured in this study. The serum levels of renin, Ang-II, and ALD in SAL group, compared with the model group, were decreased significantly, suggesting that SAL might play therapeutic effects by regulating the activity of RAAS to treat CHF *in vivo*. In addition, CCK-8 assay was used to assess the cell viability *in vitro*. The results indicated that SAL, compared with the control groups, could significantly inhibit the cell viability (more than 40 μM). Finally, 20, 10, and 5 μM of SAL was selected for the subsequent optimal concentration for further study. To directly demonstrate the protective effects of SAL on cell viability, HCS was used to qualitatively and quantitatively determine cell number, morphology, and cell viability of H9c2 cells. The results directly revealed that DOX, compared with the control group, could significantly increase the myocardial cell death, decrease the calcein AM fluorescence intensity, and increase the intensity of EthD-1 fluorescence. However, compared with the DOX group, SAL could reduce DOX-induced cardiomyocyte injury and cytotoxicity in varying degrees. Because MMP is a basic prerequisite for maintaining oxidative phosphorylation of mitochondria to produce ATP, and the stability of MMP is conducive to maintaining the normal physiological function of cells (Jayaraman, 2005), the protective effects of SAL on MMP of H9c2 cells were identified by the use of TMRE and were detected by HCS. The results showed that SAL may reduce DOX-induced mitochondrial dysfunction by affecting MMP. Thus, the potential mechanism of SAL promoting cardiomyocyte energy metabolism may be related to affecting MMP *in vitro*.

As the ATP production of mitochondrial played a pivotal role in maintaining the energy supply of cardiomyocytes and DOX could cause myocardial cell energy metabolism disorders (Pillai et al., 2010), we investigated whether SAL plays an important role in DOX-induced ATP production deficiency in H9c2 cells. The cell energy phenotype was performed to measure the metabolic phenotypes and metabolic potential of H9c2 cells. In addition, the cell mitochondrial stress was implemented to measure mitochondrial respiration function. The cell mitochondrial fuel flex test was used to determine the oxidation rate of fatty acid by measuring mitochondrial respiration of H9c2 cells in the presence or absence of fatty acid pathway inhibitors, etomoxir. Our results identified new agents to the prevention of DOX-induced cardiomyocyte injury. The results showed that treatment with SAL attenuated the energy metabolism disorder and mitochondrial dysfunction in DOX-treated H9c2 cells in terms of baseline OCR, baseline ECAR, stressed OCR and stressed ECAR, and mitochondrial respiratory function reflected in basal respiration, maximal respiration, ATP production, and H^+^ (proton) leak. Also, our results indicate that H9c2 cells’ fatty acid mitochondrial fuel usage is decreased as a result of DOX treatment compared with control group and significantly increased OCR, dependency, capacity, and flexibility of fatty acids as observed for SAL in H9c2 cells compared with the DOX group. The results suggested that SAL could be a new therapeutic agent to ameliorate DOX-induced cardiomyocyte injury and, furthermore, for the treatment of HF.

To extend our results to cell energy metabolism signaling, potential mechanisms of SAL responsible for the mitochondrial respiration function and mitochondrial energy metabolism were investigated. Mitochondrial Ca^2+^ uptake plays a pivotal role both in cell energy balance and in cell fate determination (Cristina et al., 2017). Cardiac contractility is mediated by a variable flux in intracellular calcium Ca^2+^, thought to be integrated into mitochondria *via* the MCU channel to match energetic demand. The MCU mediates high-capacity mitochondrial calcium uptake that stimulates energy production (Luongo et al., 2015). Recently, the identification of MCU and associated regulators has allowed the characterization of new physiological roles for calcium in both mitochondrial and cellular homeostasis in cytoskeletal remodeling through the modulation of ATP production (Nichols et al., 2018). In the present study, downregulation of MCU mRNA and protein expression markedly enhanced the generation of ATP decreased by DOX *in vitro*. It is noteworthy that MCU seems to have a critical role in promoting mitochondrial energy metabolism. To verify this hypothesis, we employed an MCU inhibitor ruthenium red and MCU activator spermine to specifically block or activate this pathway, to see whether the SAL still prevented H9c2 cells against DOX-induced downregulation of MCU, MICU1, and MICU2. Undoubtedly, ruthenium red cooperates with SAL to down-regulate the mRNA and protein expressions of MCU, MICU1, and MICU2 in H9c2 cells. These results verified our supposition that MCU is a critical regulatory protein in SAL-induced cardiac protection. SAL improves cardiomyocyte energy metabolism and has protective effects on mitochondrial respiratory function *via* the downregulation of MCU signaling pathway.

This study aimed to evaluate energy metabolism function and mechanism of SAL *in vivo* and *in vitro*. However, this study still has some limitations: (1) This study only tested the therapeutic effect of SAL on DOX-induced CHF model. Further studies are needed with pretreatment to determine whether SAL would be clinically useful before CHF. (2) Although several mitochondrial biomarkers were investigated in this study, the effects of SAL on the other parameters, such as real-time ATP rate, glycolytic rate, and glycolysis stress of DOX-treated H9c2 cells still need to be investigated in the future research. (3) The merit of this study is finding new active compound harboring the protective activity on cardiotoxicity. However, this study is not using positive control over the mechanism section to compare the efficacy of SAL. (4) This research tends to study the pharmacodynamics of SAL in the treatment of DOX-induced CHF. Nevertheless, the mechanism of SAL *in vivo* is insufficient. After the therapeutic effect has been confirmed, the mechanism of SAL in treating CHF should be further studied.

## Conclusion

In conclusion, this study demonstrated that SAL may be the active component of ALRP, which can treat DOX-induced CHF and ameliorate H9c2 cells from DOX-induced cardiomyocyte toxicity. The molecular mechanism responsible for cardiomyocyte activity of SAL may involve the inhibition of MCU signaling pathway, which alleviates DOX-induced mitochondrial respiratory function impairment and energy metabolism disorders to improve H9c2 cells’ dysfunction. This finding may provide evidence why ALRP has potential therapeutic effects on HF in the clinic.

## Data Availability Statement

The data used to support the findings of this study are available from the corresponding author upon reasonable request.

## Ethics Statement

The experimental protocols were approved by the Ethics Committee of the Ethics of Animal Experiments of the Fifth Medical Center of PLA General Hospital (Approval ID: IACUC-2018-010). The present study was conducted according to the recommendations of the Guidelines for the Care and Use of Laboratory Animals of the Ministry of Science and Technology of China.

## Author Contributions

J-XW and LZ performed the experiments, analyzed the data, and wrote the manuscript. H-HL, J-BW, and J-YL collected and prepared the samples. Y-XY, Y-YW, H-DC, and R-SL performed the analyses and amended the paper. Y-LZ designed the study and amended the paper.

## Funding

This work was financially supported by grants from the National Key R&D Program of China (No. 2018YFC1704500) and National Natural Science Foundation of China (81573631 and 81874365).

## Conflict of Interest

The authors declare that the research was conducted in the absence of any commercial or financial relationships that could be construed as a potential conflict of interest.

## Abbreviations

CHF, chronic heart failure; TCM, traditional Chinese medicine; ALRP, Aconiti Lateralis Radix Praeparata; SAL, salsolinol; HCS, high-content screening; MCU, mitochondrial calcium uniporter; MICU1, mitochondrial calcium uptake 1; MICU2, mitochondrial calcium uptake 2; EMRE, essential mitochondrial calcium uniporter regulator; DOX, doxorubicin; MMP, mitochondrial membrane potential; LVSP, left ventricular systolic pressure; LVEDP, left ventricle end diastolic pressure; ± LV dp/dt_max_, ± left ventricle dp/dt_max_; DH, dobutamine hydrochloride; TUNEL, terminal deoxynucleotidyl transferase (TdT)-mediated dUTP nick-end labeling; OCR, oxygen consumption rate; ECAR, extracellular acidification rate; PDH, pyruvate dehydrogenase; MDH, malate dehydrogenase; NNT, nicotinamide nucleotide transhydrogenase; BNP, brain natriuretic peptide; LDH, lactate dehydrogenase; ALD, aldosterone. RR, ruthenium red; SP, spermine.
